# CYP3A4 mediated pharmacokinetics drug interaction potential of Maha-Yogaraj Gugglu and E, Z guggulsterone

**DOI:** 10.1038/s41598-020-80595-5

**Published:** 2021-01-12

**Authors:** Sarvesh Sabarathinam, Satish Kumar Rajappan Chandra, Vijayakumar Thangavel Mahalingam

**Affiliations:** 1 Department of Pharmacy Practice, SRM College of Pharmacy, SRM Institute of Science and Technology, Kattankulathur, 603 203 Tamil Nadu India; 2 Drug Testing Laboratory, Interdisciplinary Institute of Indian System of Medicine (IIISM), SRM Institute of Science and Technology, Kattankulathur, 603 203 Tamil Nadu India

**Keywords:** Biochemistry, Biological techniques

## Abstract

Maha yogaraja guggulu (MYG) is a classical herbomineral polyherbal formulation being widely used since centuries. The aim of this study was to investigate the effect of MYG formulation and its major constituents E & Z guggulsterone on CYP3A4 mediated metabolism. In vitro inhibition of MYG and Guggulsterone isomers on CYP3A4 was evaluated by high throughput fluorometric assay. Eighteen Adult male Sprague–Dawley rats (200 ± 25 g body weight) were randomly divided into three groups. Group A, Group B and Group C were treated with placebo, MYG and Standard E & Z guggulsterone for 14 days respectively by oral route. On 15th day, midazolam (5 mg/kg) was administered orally to all rats in each group. Blood samples (0.3 mL) were collected from the retro orbital vein at 0.25, 0.5, 0.75, 1, 2, 4, 6, 12 and 24 h of each rat were collected. The findings from the in vitro & in vivo study proposed that the MYG tablets and its guggulsterone isomers have drug interaction potential when consumed along with conventional drugs which are CYP3A4 substrates. In vivo pharmacokinetic drug interaction study of midazolam pointed out that the MYG tablets and guggulsterone isomers showed an inhibitory activity towards CYP3A4 which may have leads to clinically significant interactions.

## Introduction

The use of alternative medicine such as herbal medicines, phytonutrients, ayurvedic products and nutraceuticals used widely by the majority of the patients for their primary healthcare needs. Utilization of classical herbal medicines has increased steadily for the last few decades. Most of the classical herbal medicines are chosen over conventional medicines because of their assumed safe nature^1^. Cytochrome enzymes play a crucial role in drug metabolism, and the majority of the drugs are metabolized by CYP3A4 enzyme. Inhibition/Induction of drug metabolism is a common cause of clinically important pharmacokinetic herb-drug interactions^2^. The specific substrate of CYP450 enzyme may interact with the active phytochemical compounds of herbal medication and which can diminish the metabolic clearance of a co-administered conventional medicine^3^. This issue is significantly safety concern, especially important with drugs having narrow therapeutic index such as warfarin or digoxin^4,5^.

Maha-Yograj Gugglu (MYG) tablet is widely used for the management of rheumatoid arthritis, inflammation, cholesterol-lowering activity, facial paralysis and hemorrhoids. Gugglu is naturally occurring oleoresin from *Commiphora Mukul*. Gugglu produces a synergistic effect due to the presence of bioactive compounds such as E and Z guggulsterone and guggul lipid. The hypolipidemic activity of gugglu and its various fractions has been studied in several animal models, and clinical studies E-guggulsterone and Z-guggulsterone which reported to be the main compounds play a major role in lipid-lowering activity in patients with obesity and hypercholesterolemia was found in clinical studies with crude gugglu. For the hypolipidemic action of gugglu, there was a numerous mechanism of action has been established so far. Gugglu may reduce hepatic steroid production, which is responsible for the elevation of plasma LDL cholesterol. Chief components of gugglu, *E* and *Z* guggulsterone, may elevate the binding sites of LDL cholesterol in the liver and increasing the clearance rate of LDL. Though, recent findings have proposed that the active isomer of guggulsterone are the highly effective antagonist of the farnesoid X receptor (FXR), a nuclear hormone receptor that is activated by bile acids, thus allowing increased cholesterol catabolism and excretion from the body^6^.

**Figure 1:**
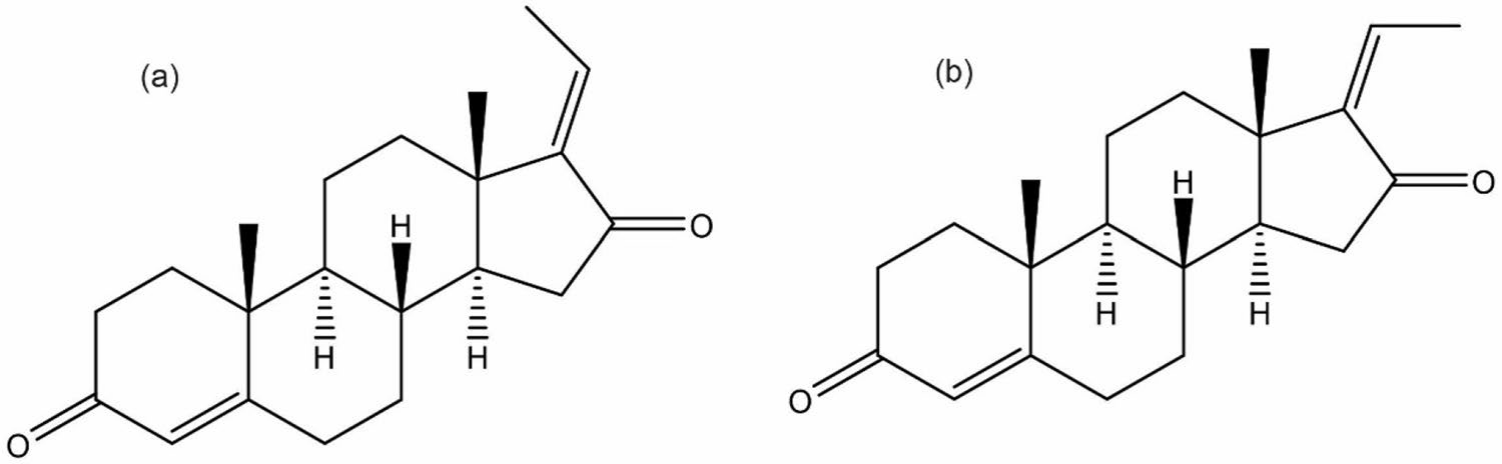
Chemical structure of E – guggulsterone (a) and Z – guggulsterone (b).

To estimate CYP3A4 mediated drug interaction, midazolam is an established sensitive probe drug due to its high fraction metabolized by CYP3A4 of 90%^7,8^. There may be a chance of occurring unintended potential herb-drug interactions through CYP3A4 inhibition/induction when herbal medicines containing E and Z guggulsterone used concomitantly. Thus, the present study was attempted to investigate the metabolism mediated pharmacokinetics drug interaction of MYG and E, Z guggulsterone (Fig. 1.) by in vitro assays and in vivo in rats. To the best of our knowledge, no studies have been conducted towards the estimation of drug interaction potential of MYG and E, Z guggulsterone by CYP450 inhibition assay and with midazolam in rat model.

## Materials and methods

### Herbal materials, chemicals and instrumentation

MYG (500 mg tablets contains 17.5 mg of guggulsterones) were purchased from AVN Ayurveda Formulations Pvt Ltd. Madurai, India. Midazolam (purity 98.0%), E & Z—guggulsterone (purity 98.0%) were also purchased from SIGMA-ALDRICH Company (St. Louis, MO). HPLC grade acetonitrile and Methanol were from Merck Company (Darmstadt, Germany). VIVID CYP450 Screening Kit and VIVID Substrates were purchased from Invitrogen Drug Discovery Solutions, USA. VIVID CYP3A4 Red (Cat. no. P2856). Ketoconazole was obtained as a gift sample from M/s Micro Labs Pvt. Ltd, Hosur, Tamil Nadu, India. All other chemicals were of analytical grade and used without further purification. All analysis was performed with liquid chromatographic system, (AGILENT technologies 1220 Infinity II LC using PDA detector). LC solution software is used to record the data. C18- 250 mm × 4.6 mm & 5 µm). other apparatus included Camag Linomat V (Switzerland) sample applicator, CAMAG automatic TLC sampler III, Remi compact laboratory high speed centrifuge, Electronic balance and PCI Stainless steel analytical ultra Sonicator bath.

### Effect of MYG and E, Z—guggulsterone on pooled rat microsomes

Liver microsomes were isolated from Adult male Sprague–Dawley rats (weight 200–250 g) based on the method described by Pandit et al.^9^. The Experimental protocol was approved by the Institutional Animal Ethical Committee (662/PO/Re/2002/CPCSEA Approval No. IAE/212/2019), SRM College of Pharmacy, SRM Institute of Science and Technology. The Protein concentrations in the liver microsomes were determined by modified biuret method (Multiskan GO Microplate Spectrophotometer, Thermo Scientific, and Waltham, MA, USA) using bovine serum albumin (BSA) as standard. BSA powder was dissolved in distilled water and diluted to a concentration of 1 μg/μl. A series of dilutions (0, 1, 2.5, 5, 10, and 20 μg/well) were made in replicates of 4 with a final volume of 100 μl. After standards and samples were diluted and transferred to the microplate, 200 μl of biuret reagent was added to each well and mixed thoroughly with repeated pipetting. The mixture was then allowed to incubate at room temperature for 20 min. Samples were mixed immediately with repeated pipetting with each addition. Colour was allowed to develop for 30 min at room temperature and the absorbance was measured at 540 nm and blanked on the water only control^10^. Screening of inhibitory activity of MYG and E, Z—guggulsterone was performed with pooled rat liver microsomes in 96 well microplate, based on the method described by Vijayakumar et al.^10^. The concentration of CYP450 was calculated using the formula (Eq. 1).

1 $$CYP450\,(mM)=\frac{\Delta {\text{APC}}-{\Delta \text{AP}}}{91}$$

PC sample, Sample Kept in the Incubator. P Sample, Sample Kept in the room temperature. Where, ΔA_PC_ is the absorbance difference of the PC sample, and ΔA_P_ is the absorbance difference of the P sample. The percentage inhibition was calculated using the following formula (Eq. 2).

2 $$\% inhibition\,of\,CYP450=\frac{\text{Blank}-\text{Test}}{\text{Blank}} \times 100$$

### High throughput fluorometric assay of CYP3A4

High throughput screening HTS assays were conducted in black 96-well microplates. Fluorescence findings were acquired from BIOTEKFLX 800 fluorescence microplate reader (BIO TEK, US) using a suitable emission/ excitation wavelength. The assay was carried out in accordance with the Invitrogen Drug Discovery Solutions, USA pre-determined guidelines. Two-fold serial dilution of the test samples were prepared and the plates were incubated for 20 min at 37 °C to determine IC_50_ values. The enzymatic reaction was initiated by the addition of a mixture of NADP + and the appropriate substrate. Plates were incubated for 10 min at 37 °C and the reaction was stopped by adding 0.5 M tris buffer. All the tests were performed in triplicate. Product formation from the fluorogenic probes was calculated from fluorescence data at seven different concentrations of the test and inhibitor (Eq. 3).^11^

3 $$\% inhibition\,of\,CYP3A4= \left(1-\frac{RFU\,in\,presence\,of\,test\,compound }{RFU\,in\,absence\,of\,test\,compound }\right) \times 100$$

where, RFU is Relative fluorescence unit.

### Pharmacokinetics study procedure in rats

Adult male Sprague–Dawley rats (200 ± 25 g body weight) were obtained from Department of Pharmacology, SRM College of Pharmacy. Before starting the experimental procedures, all the rats were kept for acclimatization for 1 week in the animal quarters under air conditioning (25 ± 1 °C) and an automatically controlled photoperiod of 12 h light daily, fed with standard rodent chow and tap water ad libitum. All experimental procedures and protocols were reviewed and approved by the Institutional animal ethics Committee and were in accordance with the Guide for the Care and Use of Laboratory Animals. Eighteen Adult male Sprague–Dawley rats were randomly divided into three groups (a total of 18 rats, n = 6).

- Group A: Placebo (Normal saline)
- Group B: MYG
- Group C: Standard E & Z guggulsterone (10.76 mg).

The MYG tablets were crushed thoroughly and 10 mg dissolved in 10 ml of water and stored at 4 °C until its administration to the rats. Each animal administered with 0.215 mg/ml concentration of MYG. Group A, Group B and Group C were treated with placebo, MYG and Standard E & Z guggulsterone solution for 14 days respectively by oral gavage. 15th day a Midazolam (5 mg/kg) in carboxy methyl cellulose—Na solution was administered orally to all rats in each group. Blood samples (0.3 mL) were collected from the retro orbital vein into heparinized 1.5 ml polythene tubes at 0.25, 0.5, 0.75, 1, 2, 4, 6, 12 and 24 h of each rat were collected. During the seventh blood collection, the rats were treated with 2 ml of normal saline in order to restore blood capacity quickly. The samples were immediately centrifuged at 4000×*g* for 10 min and the plasma was stored at − 20 °C until analysis.

### Chromatographic conditions and quantification of E & Z guggulsterone by HPTLC method

MYG tablet was standardized by using Camag HPTLC and the E & Z guggulsterone was used as standard. HPTLC was performed on band width 6 mm with a Camag 100 µl sample syringe (Hamilton, Bonaduz, Switzerland) on Silica gel precoated aluminum 60 F254 plates (20 cm × 10 cm with 250 μm thickness). Solution of MYG tablet (10 mg/ml) was prepared by using methanol and the solution was filtered through nylon syringe filter (0.45 µm). Same procedure was followed to make the solution of E & Z guggulsterone (1 mg/ml). Different volume of test and standard solutions were applied by Linomat V (Switzerland) sample applicator. The developed chamber was saturated using (Acetone: Toluene (19:1 v/v)) as a mobile phase. Then the plates were removed, dried at room temperature and scanned under UV-254 nm. Densitometric scanning is used to estimate the quantity of E & Z guggulsterone in MYG tablet.

### Chromatographic conditions for HPLC method

In this chromatographic system, PDA detector is used (AGILENT technologies 1220 Infinity II LC). LC solution software is used to record the data. C18-250 mm × 4.6 mm & 5 µm, using a mobile phase containing Acetonitrile: water (ACN) (70:30% v/v). A flow of 1 ml/min, injection volume of 10 µl, detection wavelength of 220 with the run time of 5mins and instrument was functioned at ambient temperature. The peak purity was checked with the photodiode array detector. Quantification of compound is determined by peak-area method. The quantification of midazolam & alpha hydroxy midazolam was performed using HPLC–MS techniques by the peak-area method. SIM mode is performed to determine the target ions (m/z 326 for midazolam, m/z342.50 for alpha hydroxy midazolam and m/z 237 for internal standards). The quantification was estimated with a signal-to-noise ratio of > 10, a precision of RSD < 20% and RE < 15% verified with five consecutive replicates. Limit of Detection (LOD) were 0.17 μg/ml^−1^ for Midazolam and 0.12 μg/ml^−1^ for alpha hydroxy midazolam, Limit of Quantification (LOQ) was 0.85 μg/ml^−1^ for Midazolam and 0.6 μg/ml^−1^ for alpha hydroxy midazolam. The linear range used was 5.0–1000 ng/mL in rat plasma for all analytes. The outcome of chromatographic validation showed that analytical methods were suitable for this analysis.

### Statistical analysis

The results were presented as the mean ± standard deviation. IC_50_ values concentration required to cause a 50% inhibition in enzyme activity were obtained using mean enzyme activity versus inhibitor concentration curves using GraphPad prism Version 5.01 (GraphPad Prism Software Inc.,USA). Using the plasma concentration and time curves all the Pharmacokinetics parameters were estimated by non-compartmental analysis using Winnonlin software. Comparisons between the groups A, B & C were performed by analysis of variance (One Way ANOVA.

## Results

Standardization of MYG was performed through HPTLC using E & Z guggulsterone as a standard. The calibration curve for Standard E & Z guggulsterone showed good linearity (2–10 µL). The correlation coefficient for (r^2^) was 0.995 and 0.994 for E-guggulsterone and Z-guggulsterone respectively, which showed good correlation between drug content and peak area. The linear equation for E-guggulsterone is Y = 59.617x + 15.533 for Z- guggulsterone Y = 67.017x + 73.533. The peak of E- guggulsterone and Z- guggulsterone in the MYG tablet was identified by comparing the R_f_ value of reference standard at 0.61 for E-guggulsterone and 0.23 for Z-guggulsterone. The findings have been adequate and suitable for further quantitative study. The amount of E-guggulsterone & Z-g uggulsterone in the MYG tablet was found to be 4.99% (w/w) and 3.95% (w/w) respectively.

**Figure 2:**
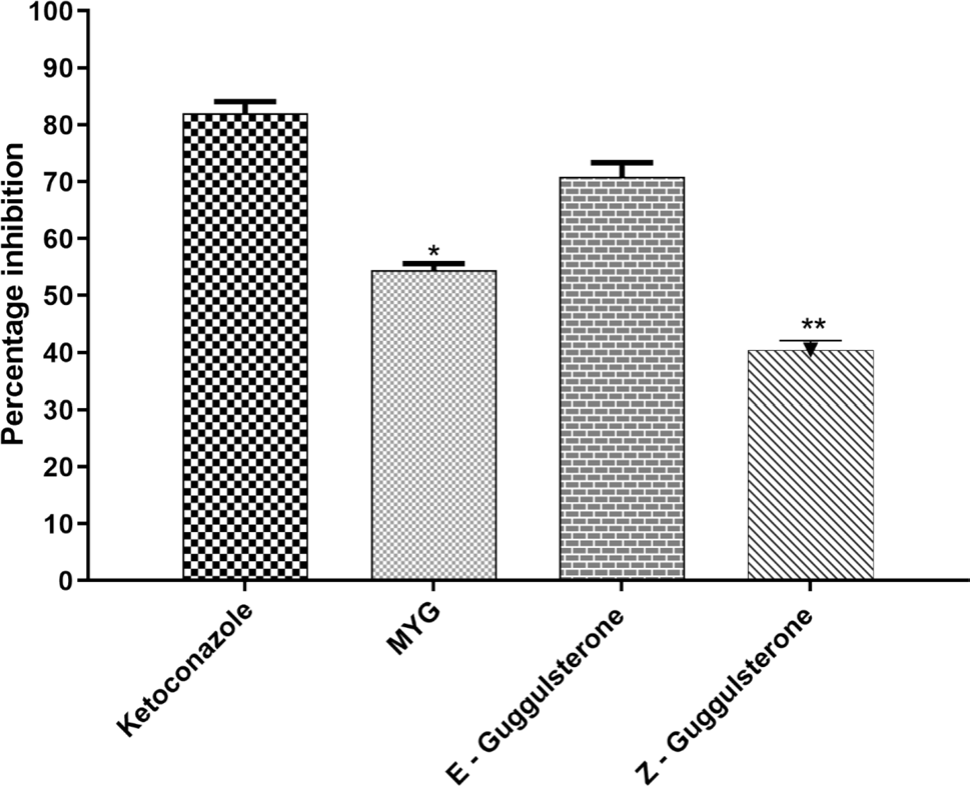
Percentage inhibition of MYG and guggulsterone isomers versus ketoconazole. Values are expressed in mean ± SEM; n = 3; ANOVA followed by Dunnett’s multiple comparison Test. Level of significance at *P < 0.05, *P* < 0.01**.

The concentration of protein in pooled rat liver microsome was found to be 7.6 mg/ml. CYP450-CO Complex assay was used to assess the inhibitory potential of MYG and guggulsterone isomers. The CYP450 concentration of the diluted microsome sample was calculated and was found to be 0.442 nmol/mg of protein. E-guggulsterone dissolved in DMSO showed higher percentage of inhibition (70.88 ± 2.45%) but MYG tablets (54.44 ± 1.16%, *p* < 0.05*) and Z-guggulsterone (40.44 ± 1.67%, *p* < 0.01**) shown significant difference in the percentage of inhibition when compared to positive control (Ketoconazole) (Fig. 2).

**Figure 3:**
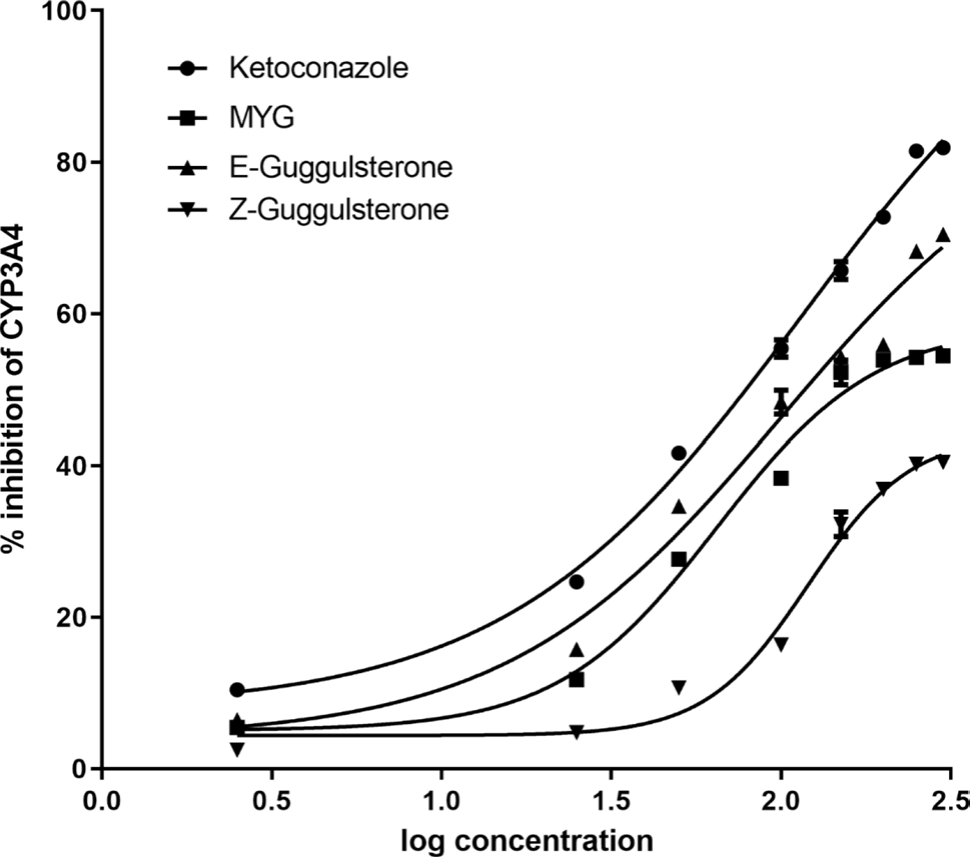
Concentration dependent inhibitory effect of MYG, guggulsterone isomers and ketoconazole on CYP3A4.

Various concentrations ranging from 2.5 to 250 µg/ml of MYG, E-guggulsterone and Z-guggulsterone were evaluated for their ability to affect the biotransformation of conventional drugs when administered concomitantly. MYG and guggulsterone isomers showed CYP3A4 mediated inhibitory activity in concentration dependent manner (Fig. 3). All samples were assayed in triplicate, the endpoint mode was selected and IC_50_ values were calculated Table 1. IC_50_ values of E-guggulsterone dissolved in DMSO on CYP3A4 was 03.96 ± 1.04 µg/ml but it did not show significant difference when compared to positive control (01.74 ± 0.66 µg/ml).

**Table 1:** The IC_50_ (µg/ml) value of MYG, E-guggulsterone, Z-guggulsterone and positive control.

Test samples	CYP3A4 IC_50_ (µg/ml)
MYG	11.43 ± 2.12*
E-guggulsterone	03.96 ± 1.04
Z-guggulsterone	24.08 ± 0.48**
Ketoconazole	01.74 ± 0.66

Level of significance at *P < 0.05, **P < 0.01.

**Figure 4:**
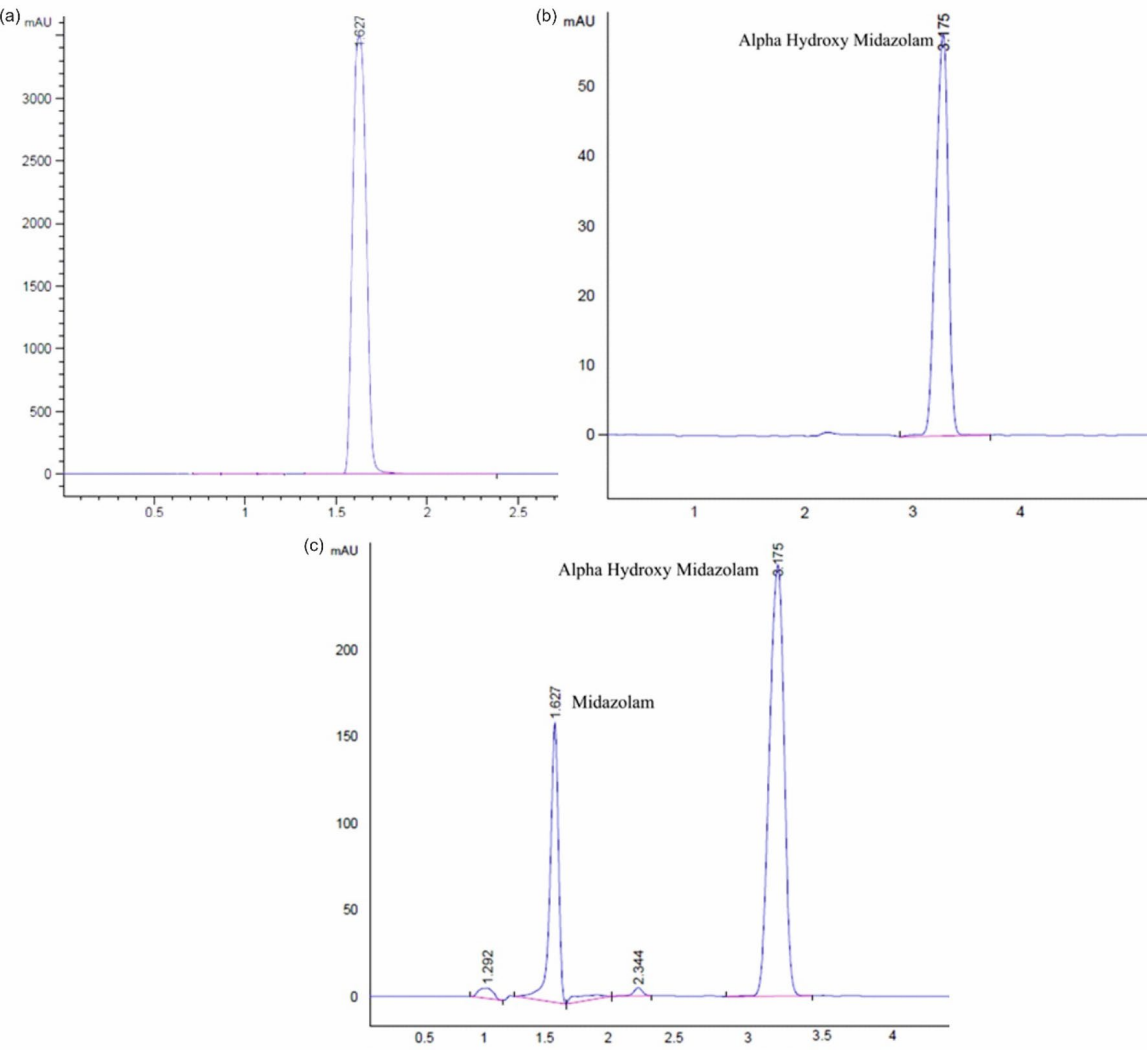
(**a**) HPLC Chromatogram of Standard midazolam, (**b**) HPLC Chromatogram of alpha hydroxy midazolam, (**c**) HPLC Chromatogram of midazolam (50 µg/ml), and alpha hydroxy midazolam (49.6 µg/ml) in rat plasma.

In the present study, RP-HPLC–MS was used for the method development and validation of CYP3A4 probe drug (midazolam) in rat plasma after oral administration. Good chromatographic separation of midazolam and alpha hydroxy midazolam was achieved (Fig. 4). The Drug-Drug interaction (DDI) effect of MYG tablet and guggulsterones was determined comparing the midazolam pharmacokinetics profiles (Table 2). The clearance of midazolam in Group A [0.10 ± 0.02 (ng/ml)/h] was significantly decreased (*p* < *0.05*) in Group B [0.07 ± 0.01 (ng/ml)/h] and Group C [0.05 ± 0.010 (ng/ml)/h]. In Group B and Group C, significant elevation of AUC (144.5 ± 4.6 μg/ml*h, *p* < 0.01) and C_max_ (31.52 ± 2.34 μg/ml, *p* < 0.05) was found when compared to Group A.

**Table 2:** Pharmacokinetic parameters of midazolam in rat plasma.

Parameters	Group A (n = 6) Placebo	Group B (n = 6) MYG	Group C (n = 6) E & Z guggulsterone
T_max_ (h)	0.09 ± 0.01	0.08 ± 0.012	0.07 ± 0.007
C_max_ (μg/ml)	25.29 ± 12	31.52 ± 2.34*	35.5316 ± 1.57^ɸ^
AUC_0-t_ (μg/ml*h)	109.22 ± 32	144.5 ± 4.6**	169.4 ± 3.52^ɸɸ^
AUC_0-inf_ (μg/ml*h)	110.91 ± 0.1	145.9 ± 3.7***	169.9 ± 4.01^ɸɸɸ^
T_1/2_ (h)	1.45 ± 0.1	1.5 ± 0.01	2.01
CL/F (ng/ml)/h	0.10 ± 0.02	0.07 ± 0.01*	0.05 ± 0.010^**ɸ**^

Values are expressed in mean ± SD; (*) *P* < 0.05 Group A vs Group B; (ɸ) *P* < 0.05 Group A Vs Group C.

*T*_*max*_ time required to achieve maximum plasma concentration; *C*_*max*_ maximum plasma concentration; *AUC*_*(0–24)*_ area under concentration–time curve extrapolated to 24 h; *AUC*_*(0–∞)*_ area under concentration–time curve extrapolated to infinity; *CL/F* total body clearance; *t½* half-life.

**Figure 5:**
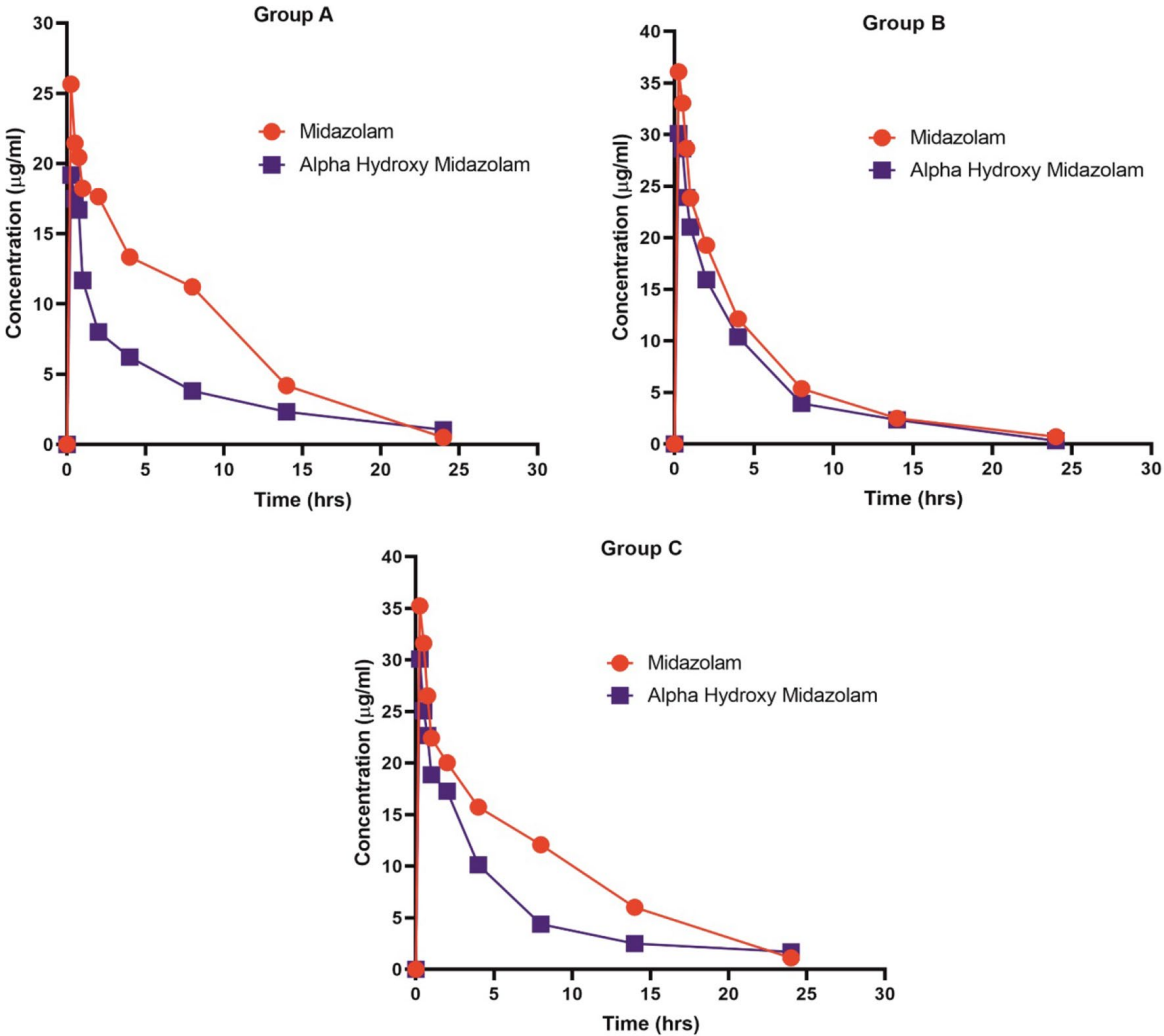
Mean plasma concentrations Vs Time curves of midazolam and alpha hydroxyl midazolam in Group A, Group B and Group C.

Similarly, midazolam metabolite (alpha hydroxy midazolam) in Group B showed significant elevation in AUC (113 ± 4.48 μg/ml*h, *p* < 0.01) and C_max_ (32.51 ± 0.63 μg/ml, *p* < 0.05) when compared with Group A and Group C. Unlike, midazolam pharmacokinetics, alpha hydroxy midazolam shown significant decrease in Group B and C when compared to Group A (*p* < 0.05) in T_max_ (Table 3). The results indicated that the metabolism of midazolam in Group B and C was evidently slowed down hence MYG tablet and its major phytoconstituents studied (E & Z guggulsterone) had the potential to inhibit rat hepatic CYP450 activity in vivo. Mean plasma concentration vs Time curves of midazolam and alpha hydroxy midazolam in Group A, Group B and Group C were presented in Fig. 5.

**Table 3:** Pharmacokinetic parameters of alpha hydroxy midazolam in rat plasma.

Parameters	Group A (n = 6) Placebo	Group B (n = 6) MYG	Group C (n = 6) E & Z guggulsterone
T_max_ (h)	0.15 ± 0.9	0.08 ± 0.003*	0.07 ± 0.004^ɸ^
C_max_ (μg/ml)	20.29 ± 44	32.51 ± 0.63*	32.95 ± 9.5^ɸ^
AUC_0-t_ (μg/ml*h)	99.15 ± 43	113 ± 4.48**	128.6 ± 4.6^ɸɸ^
AUC_0-inf_ (μg/ml*h)	99.16 ± 87	113 ± 4.52***	129.5 ± 2.3^ɸɸɸ^
T_1/2_ (h)	1.25 ± 0.2	1.45 ± 0.5	1.55
CL/F (ng/ml)/h	0.09 ± 0.1	0.007 ± 0.2	0.007 ± 0.1

Values are expressed in mean ± SD; (*) *P* < 0.05 Group A vs Group B; (ɸ) *P* < 0.05 Group A Vs Group C.

*T*_*max*_ time required to achieve maximum plasma concentration; *C*_*max*_ maximum plasma concentration; *AUC*_*(0–24)*_ area under concentration–time curve extrapolated to 24 h; *AUC*_*(0–∞)*_ area under concentration–time curve extrapolated to infinity; *CL/F* total body clearance; *t½* half-life.

## Discussion

In the contemporary state, there has been a rapid rise in the use of alternative medicines especially herbal medicines for various disorders, Resins from the Commiphora species have been widely used for the management of arthritis, inflammation, cholesterol lowering activity, obesity, hemorrhoids and gastrointestinal disturbance^12,13^. Guggulsterone is one of the major bioactive compounds in commiphora species and also in MYG tablets, which is act as inhibitor on Farnesoid X receptor resulting in reduced lipid levels^14^. Even though there was no specific drug toxicity or mortality reported for alternative medicines, there is still risk of herb-drug interactions.

In the present study, an approach was made to evaluate the interaction potential of MYG and isomers of guggulsterone on CYP450 through rat liver microsomes and high throughput fluorescence screening assay for CYP3A4. The use of healthy human subjects is considered as ideal approach to generate clinically significant pharmacokinetic herb-drug interaction information on MYG and guggulsterones. However, due to the risk associated with such studies in humans and the rigorous ethical and regulatory requirements, the use of in vitro and animal models has become the best alternative^15^. In both in vitro assays, E-guggulsterone had equal inhibitory potential as like positive control because there was no significant difference in percentage of CYP450 inhibition and IC_50_ values of CYP3A4 of the positive control ketoconazole. As evident from in vitro study, E-guggulsterone is a major phytomarker has significant interaction potential with CYP3A4 enzymes when compared to extracts of MYG tablets. In vitro CYP450 assays offer an accurate, relatively inexpensive, first stage assessment tool to determine the potential for herb-drug interactions. They are useful for initial risk assessment of herbal medicines capable of causing adverse herb-drug interactions when taken concomitantly with allopathic drugs metabolized by the same enzyme^16^. We have selected CYP3A4 probe drug, midazolam for the estimation of drug interaction potential of MYG tablets and guggulsterone isomers for in vivo method.

The preclinical pharmacokinetic drug interaction study revealed that the significant increases in AUC and slight increase in C_max_ of midazolam in the MYG Tablets and guggulsterone isomers treated groups (Group B & C) were indicative of enhanced bioavailability of the drug. According to Jambhekar and Breen^17^, AUC and C_max_ are used in the measurement of the extent of drug bioavailability which inter alia is dependent on first-pass effect. Therefore, the higher values of AUC and C_max_ in the MYG Tablets and guggulsterone treated rats were indicative of enhanced systemic availability of midazolam and possible inhibition of CYP450 enzymes involved in the metabolism of midazolam i.e. CYP3A4 and CYP2C11 both in the intestine and liver. Simultaneous administration of this herb with drugs which are metabolized by CYP3A4 enzyme may lead to unintended drug interactions and severe adverse drug events that may impede drug adherence. In order to prevent or predict interactions, assessment of which herbal medications a patient is taking should be part of the treatment planning or therapeutic decision making stage.

## Conclusion

The findings from the in vitro study proposed that the MYG tablets and its guggulsterone isomers have drug interaction potential when consumed along with conventional drugs which are CYP3A4 substrates. The pharmacokinetic study of midazolam confirmed the inhibitory activity of MYG tablets and its major active constituents such as E & Z guggulsterone towards CYP450 enzymes in the rat model. Specifically, the clearance of midazolam decreased while the C_max_ increased, indicating MYG and guggulsterones affected the midazolam pharmacokinetics. Therefore, to avoid clinically significant herb-drug interaction, caution should be taken when MYG or guggulsterones are co-administered with other allopathic drugs metabolized by CYP3A4.

## Data Availability

The authors confirm that the data supporting the findings of this study are available within the article and its supplementary materials.
